# Diurnal Regulation of Cellular Processes in the Cyanobacterium *Synechocystis* sp. Strain PCC 6803: Insights from Transcriptomic, Fluxomic, and Physiological Analyses

**DOI:** 10.1128/mBio.00464-16

**Published:** 2016-05-03

**Authors:** Rajib Saha, Deng Liu, Allison Hoynes-O’Connor, Michelle Liberton, Jingjie Yu, Maitrayee Bhattacharyya-Pakrasi, Andrea Balassy, Fuzhong Zhang, Tae Seok Moon, Costas D. Maranas, Himadri B. Pakrasi

**Affiliations:** a Department of Biology, Washington University, St. Louis, Missouri, USA; b Department of Energy, Environmental, and Chemical Engineering, Washington University, St. Louis, Missouri, USA; c Department of Chemical Engineering, The Pennsylvania State University, University Park, Pennsylvania, USA

## Abstract

*Synechocystis* sp. strain PCC 6803 is the most widely studied model cyanobacterium, with a well-developed omics level knowledgebase. Like the lifestyles of other cyanobacteria, that of *Synechocystis* PCC 6803 is tuned to diurnal changes in light intensity. In this study, we analyzed the expression patterns of all of the genes of this cyanobacterium over two consecutive diurnal periods. Using stringent criteria, we determined that the transcript levels of nearly 40% of the genes in *Synechocystis* PCC 6803 show robust diurnal oscillating behavior, with a majority of the transcripts being upregulated during the early light period. Such transcripts corresponded to a wide array of cellular processes, such as light harvesting, photosynthetic light and dark reactions, and central carbon metabolism. In contrast, transcripts of membrane transporters for transition metals involved in the photosynthetic electron transport chain (e.g., iron, manganese, and copper) were significantly upregulated during the late dark period. Thus, the pattern of global gene expression led to the development of two distinct transcriptional networks of coregulated oscillatory genes. These networks help describe how *Synechocystis* PCC 6803 regulates its metabolism toward the end of the dark period in anticipation of efficient photosynthesis during the early light period. Furthermore, *in silico* flux prediction of important cellular processes and experimental measurements of cellular ATP, NADP(H), and glycogen levels showed how this diurnal behavior influences its metabolic characteristics. In particular, NADPH/NADP^+^ showed a strong correlation with the majority of the genes whose expression peaks in the light. We conclude that this ratio is a key endogenous determinant of the diurnal behavior of this cyanobacterium.

## IMPORTANCE

Cyanobacteria are photosynthetic microbes that use energy from sunlight and CO_2_ as feedstock. Certain cyanobacterial strains are amenable to facile genetic manipulation, thus enabling synthetic biology and metabolic engineering applications. Such strains are being developed as a chassis for the sustainable production of food, feed, and fuel. To this end, a holistic knowledge of cyanobacterial physiology and its correlation with gene expression patterns under the diurnal cycle is warranted. In this report, a genomewide transcriptional analysis of *Synechocystis* PCC 6803, the most widely studied model cyanobacterium, sheds light on the global coordination of cellular processes during diurnal periods. Furthermore, we found that, in addition to light, the redox level of NADP(H) is an important endogenous regulator of diurnal entrainment of *Synechocystis* PCC 6803.

## INTRODUCTION

Oscillatory behaviors are observed in eukaryotic organisms such as fungi, algae, plants, and animals (1–4). Among the prokaryotes, cyanobacteria (5–7) are the only eubacteria that exhibit robust oscillatory behavior. The most common form of oscillation is circadian rhythm, with an approximate period length of 24 h, in order to respond to daily environmental changes. *Synechocystis* sp. strain PCC 6803 (hereafter, *Synechocystis* PCC 6803), a unicellular and nondiazotrophic cyanobacterium, has been widely used as a model strain to study cellular physiology, as well as for metabolic engineering applications (8–10). Similar to other cyanobacteria, *Synechocystis* PCC 6803 carries out photosynthesis and glycogen synthesis in the light and respiration and glycogen degradation in the dark (11, 12). However, unlike other well-studied cyanobacteria, only 9% of *Synechocystis* PCC 6803 genes apparently exhibit circadian-cycle-regulated oscillatory temporal expression patterns (10). In a recent study, Beck et al. further substantiated this claim by conducting gene expression measurements during light/dark (L/D) cycles followed by constant L/D conditions (9). Interestingly, they determined that many genes exhibit cyclic gene expression patterns controlled by diurnal cycles (9). However, the interplay between such rhythmic changes and cellular physiology/metabolism remains largely unexplored. Furthermore, less well studied are the presence of any endogenous determinant of the diurnal behavior and its correlations with metabolic homeostasis and diurnal entrainment (13, 14).

*Synechocystis* PCC 6803 possesses various metabolic pathways/subsystems, and interactions among them vary to respond to different environmental conditions (15). It can perform autotrophic, mixotrophic, photoheterotrophic, and heterotrophic growth, and fluxes through central carbon metabolism under such varying conditions have been estimated by metabolic flux analysis (16–18). On the basis of these studies, we know that in the presence of light, *Synechocystis* PCC 6803 activates the Calvin cycle to supply fixed carbon for various metabolic processes. In addition, under photosynthetic conditions, the conversion between 3-phosphoglyceric acid and glyceraldehyde phosphate (GAP) is in the reductive direction and is catalyzed by the GAP2 isoform (19). Under heterotrophic conditions, glucose/glycogen is utilized through glycolysis and the oxidative pentose phosphate pathway (OPPP) to supply carbon skeletons and energy/reducing power for growth, respectively (20). However, the changes in the metabolic and/or physiological state of *Synechocystis* PCC 6803 that occur during a diurnal L/D cycle have yet to be reported.

In the present study, we explored the global transcriptional changes in *Synechocystis* PCC 6803 during a diurnal cycle and correlated them with cellular physiology and metabolism via flux balance analysis (FBA) (21) and measurements of important physiological parameters. Transcripts of nearly 40% of the genes exhibited cyclic behavior that revealed two distinct transcriptional networks of coregulated oscillatory genes. These networks elucidate the regulation of the cellular processes at the end of the dark period in order to use light efficiently at the beginning of the light period. Using *i*Syn731, our previously developed genomic-scale metabolic model of *Synechocystis* PCC 6803 (22), we analyzed the details of diurnal changes in different biochemical pathways. Furthermore, the similarity of the observed trend in the NADPH/NADP^+^ ratio to the corresponding trends in the majority of the genes whose expression peaks in the light indicates that this ratio is a key endogenous regulator of the oscillatory patterns of these genes.

## RESULTS

### Identification of oscillatory genes.

To understand the temporal variation over L/D cycles, we analyzed the changes in differential transcript abundance in *Synechocystis* PCC 6803 cells by using a genomic-scale microarray. Over a 48-h time course (or two L/D cycles), samples from cells grown in alternating 12-h L/D cycles were collected every 2 h, starting with time point D1 (i.e., after 1 h of darkness). In most cases, genes showed similar oscillation patterns in two consecutive 24-h periods, thus demonstrating the robustness of their respective expression profiles (see Fig. S1 to S6 in the supplemental material). On the basis of our analyses, 1,345 genes (equivalent to ~39% of the genes represented on the microarray) with cycling behavior were identified (see Table 1; Data Set S1 for more information).

**TABLE 1:** Peaks in the expression of all cycling genes in *Synechocystis* PCC 6803 across various functional categories^a^

Functional category (no. of genes)	No. of genes with peak expression at:											
	D1	D3	D5	D7	D9	D11	L1	L3	L5	L7	L9	L11
Amino acid metabolism (42)	4	0	0	1	1	8	14	9	2	1	1	1
Biosynthesis of cofactors (62)	4	1	0	5	1	14	22	9	3	2	0	1
Cell envelope (37)	0	0	0	0	1	5	13	13	1	4	0	0
Cellular processes (27)	1	1	0	2	1	7	11	3	0	1	0	0
Central intermediary metabolism (13)	0	0	0	0	0	4	4	4	0	0	1	0
DNA replication, restriction, modification and repair (28)	2	0	0	1	1	11	7	4	0	1	1	0
Energy metabolism (41)	2	0	0	0	2	6	19	9	0	1	1	1
Fatty acid, phospholipid, sterol metabolism (13)	0	0	0	0	0	1	9	1	0	0	0	2
Other categories (92)	5	0	3	7	10	24	15	19	2	1	3	3
Photosynthesis and respiration (106)	1	1	0	1	1	5	14	69	1	4	5	4
Purine, pyrimidines, nucleosides and nucleotides (14)	0	0	0	1	0	3	7	3	0	0	0	0
Regulatory functions (62)	5	2	2	5	5	21	12	7	0	2	1	0
Transcription (15)	1	0	0	2	1	0	5	4	1	1	0	0
Translation (53)	1	0	0	2	1	10	27	10	1	1	0	0
Transport and binding proteins (70)	2	2	2	5	3	25	16	8	3	0	3	1
Unassigned and hypothetical (670)	31	7	15	51	44	145	173	113	32	25	16	18

a Functional categories were assigned on the basis of the CyanoBase database (http://bacteria.kazusa.or.jp) The values were calculated as described in Materials and Methods and averaged over two 24-h time courses.

**FIG 1:**
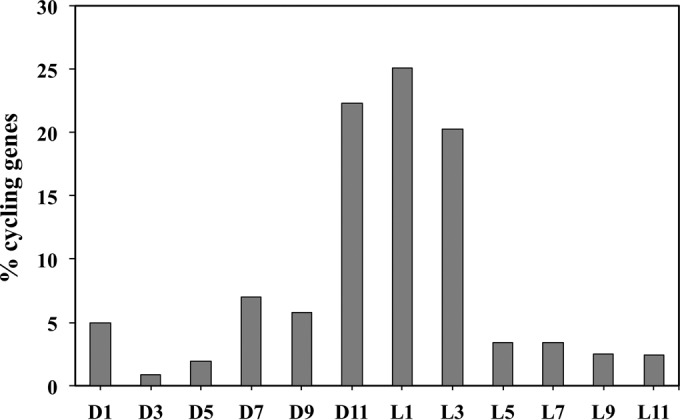
Cycling of gene expression in *Synechocystis* PCC 6803. Almost two-third of the cyclic genes peaked at the end of the dark period (i.e., D11) or the early light period (i.e., L1 and L3). The time point of the peak expression of any gene was calculated by averaging its expression levels over two consecutive diurnal cycles. Percentages are of all cycling genes.

The majority of the genes from all but three COG classifications, (i) DNA replication, restriction, modification, and repair, (ii) regulatory functions, and (iii) transport, were maximally expressed during the light period (Table 1; Fig. 1; see Fig. S1 to S4 in the supplemental material). Among the cycling genes whose expression peaks in the light, 46% and 35% had maximal transcript levels at L1 or L3, respectively (Table 1; Fig. 1). This set presumably includes genes under strict diurnal control. Interestingly, for 54% of the genes upregulated during the dark period, maximal expression occurred at D11, whereas only about 1% of such genes were highly expressed at L11. Overall, 20% more genes were maximally expressed in the light period than in the dark period. Overall, *Synechocystis* PCC 6803 cells exhibited limited transcriptional activity in the dark period. However, *Synechocystis* PCC 6803 carried out important transport, binding, and regulatory activities at D11 (preceding the transition from dark to light), presumably in anticipation of light in the following period to make the best use of the available resources.

### Network analysis.

To visualize genes with similar temporal expression profiles, we generated a coexpression network with the Cytoscape program (23, 24). Remarkably, this process yielded two separate networks: one that contains 781 genes whose expression peaks in the light period and the other with 522 genes whose expression peaks in the dark (see Movie S1 in the supplemental material). Therefore, these two networks contain a total of 1,303 genes, about 37% of the genes in the microarray (Table 1). Genes showing similar expression profiles over two consecutive L/D cycles clustered together. In addition, 42 cyclic genes were not part of either of these two networks mainly because of the cutoff set for this network analysis (see Materials and Methods for details).

Excluding the genes with hypothetical or unknown functions, we focused on how the annotated genes behaved in two consecutive L/D cycles. As shown in Table 1, the network whose expression peaks in the light contains a majority of the genes corresponding to photosynthesis; respiration; translation; energy metabolism; cell envelope development; amino acid biosynthesis; fatty acid, phospholipid, and sterol metabolism; transcription; and purine, pyrimidine, nucleotide, nucleoside, and central intermediary metabolism. On the other hand, genes associated with transport, regulatory functions and replication, restriction/modification, recombination, and repair of DNA constitute the network whose expression peaks in the dark. In addition, genes for the biosynthesis of cofactors, prosthetic groups, and carriers and other cellular processes (such as protein and peptide secretion, cell division, detoxification, and chemotaxis) are almost equally distributed across these two networks. As mentioned above, genes from the network whose expression peaks in the light were upregulated at the beginning of the light period in all cases but one (i.e., photosynthesis and respiration). It is noteworthy that the majority of the genes from photosynthesis and respiration did not peak at L1; instead, they reached their peak expression levels later, at L3. Contrary to the genes whose expression peaks in the light, the majority of the genes from the network whose expression peaks in the dark were upregulated in the very last phase (i.e., D11) of the dark period. This suggests that switching from darkness to light leads to the maximal expression of many genes whose expression peaks in the dark at D11 in anticipation of the physiological state of *Synechocystis* PCC 6803 in the upcoming light period.

### Dynamics of important cellular processes under diurnal rhythm. (i) Light harvesting.

In *Synechocystis* PCC 6803, phycobilisomes are the major light-harvesting antenna (25). It has been estimated that phycobilisomes account for up to 50% of the total protein pool in cyanobacterial cells, and thus, the assembly/disassembly and regulation of these complexes represent a considerable investment of cellular resources (26). Consistent with a need for tight control of phycobilisomes, our analysis of the diurnal behavior of phycobilisome-related genes showed that the majority of these genes were overall similarly controlled, with high levels of expression during the earlier light period (mostly at L3) and much lower levels in the late light period (i.e., L5 and beyond) and all through the dark period (see Fig. S1A in the supplemental material). In fact, of the genes in our experimental data set, *cpcA* (*sll1578*) and *cpcB* (*sll1577*) showed the greatest change in expression levels between light and dark. Notable among these genes was a set that showed similar cyclic behavior with lower amplitudes. These are *apcD* (*sll0928*), encoding a second copy of the APC β subunit, one of the isoforms of *cpcF* (*sll1051*), encoding phycocyanobilin lyases, and one of the isoforms of *cpcG* (*slr2051*), encoding the rod-core linker. The strongly cycling genes *cpcA*, *cpcB*, and *cpcC* (*sll1579* and *sll1580*) are organized in the same operon, while *cpcF* (*sll1051*) and *cpcG* (*slr2051* and *sll1471*) are distantly located. Interestingly, the *cpcD* (*ssl3093*) gene, encoding a rod linker and not located in the *cpcABC* operon, maintains the same cycling pattern as the rod linker gene *cpcC.*

Under environmental stresses such as nitrogen or sulfur starvation, cyanobacteria utilize phycobilisomes as a nutrient source through a degradation process that leads to yellowing or bleaching of the culture. The *nbl* (nonbleaching) genes are involved in this process, with *nblA* interacting directly with the phycobilisome, possibly causing rod instability and the beginning of the disassembly process. We found that the expression of the *nblA1* (*ssl0452*) and *nblA2* (*ssl0453*) genes was higher in the dark, peaking at D11, and much lower in the light. The NblB protein sequence is similar to that of the phycocyanobilin lyase that functions in the chromophore attachment to the apoprotein and has been proposed to interact with the chromophores in the degradation process. Similar to *nblA*, *nblB2* (*slr1687*) was also more highly expressed in the dark (with a peak at D7) than in the light (see Fig. S1A in the supplemental material). These data are consistent with a model in which phycobilisomes are actively degraded during the late dark period and resynthesized mainly in the early light period.

### (ii) Photosynthesis and respiration.

**FIG 2:**
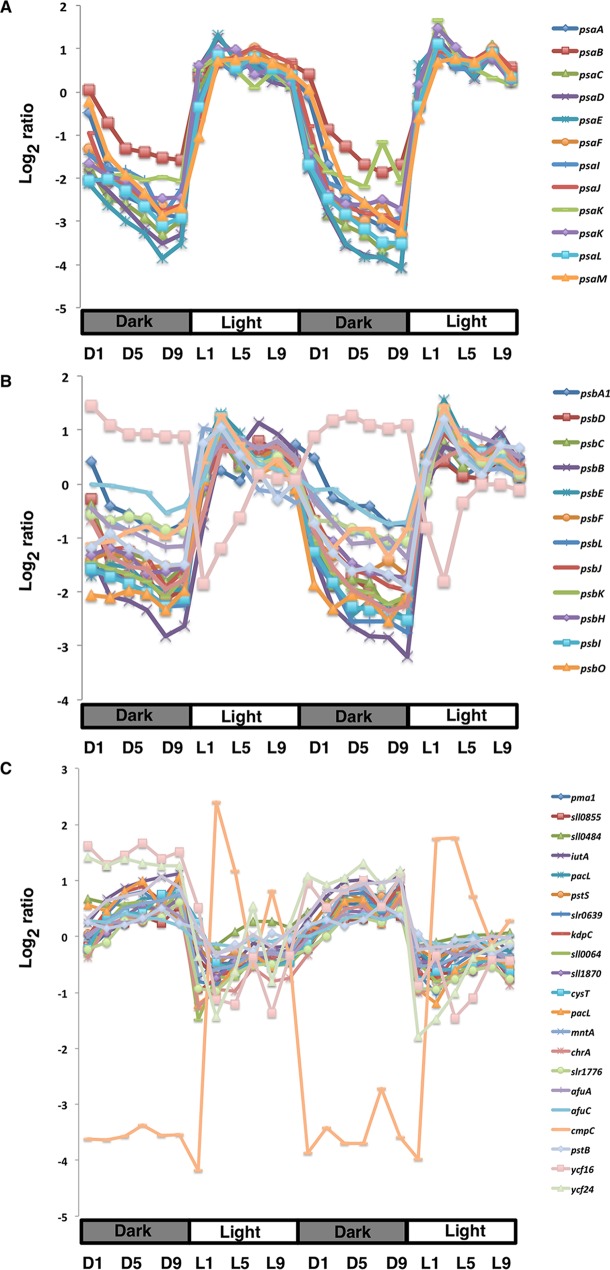
Expression profiles of genes with cyclic patterns involved in PSI (A), PSII (B), and membrane transport (C). L/D cycles are indicated as gray and white bars below the *x* axis, respectively. The log_2_ ratios of transcript abundance to the pooled sample control are plotted on the *y* axis.

The majority of the photosystem I (PSI) and photosystem II (PSII) gene transcripts were highly upregulated during the early light period and significantly downregulated during the dark period (Fig. 2A and B). One notable exception was the *slr1739* gene (encoding the Psb28-2 protein), which was upregulated during the entire dark period. This finding calls into question the role of this enigmatic protein in PSII. Most of the *cytb_6_f* transcripts behaved similarly; they are upregulated in the early light period and downregulated in the dark. Notable exceptions were the three different *petC* transcripts for the Rieske iron-sulfur center protein. One of them, *petC1* (*sll1316*) behaved similarly to the transcripts for the other structural proteins of this complex. In contrast, transcripts for *petC2* (*slr1185*) and *petC3* (*sll1182*) appeared to be noncyclic. An earlier study indicated that PetC1 is the major Rieske protein in the *cytb_6_f* complex in *Synechocystis* PCC 6803 (27).

In *Synechocystis* PCC 6803, two alternate donors in the thylakoid lumen transfer electrons from the *cytb_6_f* complex to the PSI complex (see Fig. S1C in the supplemental material). Plastocyanin (*petE* gene) is a copper protein, and cyt*c*_553_ (*petJ* gene) is a heme protein. Both transcripts were present in cells grown in BG11 (0.3 µM Cu) (28) and were significantly upregulated at L3 and downregulated after that time. There are four *petC* genes for ferredoxin-like proteins in *Synechocystis* PCC 6803. The transcript for one of them, *fed1* was upregulated during the light period and highly downregulated in the dark (see Fig. S2A in the supplemental material). In contrast, the transcript for *slr0150* seemed to be upregulated in the early dark period. The transcript for *slr1643*, encoding FNR, was highly upregulated at the beginning of the light period and quickly decayed beginning at L5. All ATP synthase genes were upregulated during the early light period and significantly downregulated during the dark period (see Fig. S1B).

Overall, as expected, all transcripts associated with photosynthetic electron transport and ATP synthesis were upregulated during the early light period and downregulated during darkness. However, the behavior of key genes involved in respiration was more intriguing. Contrary to the current understanding, both NADH dehydrogenase and cytochrome *c* oxidase genes were upregulated at the beginning of the light period, which further alludes to the intricate topology and cross talk of respiratory and photosynthetic electron transport chains (see Fig. S2B and C in the supplemental material for details).

### (iii) Central carbon metabolism.

The carbon concentration mechanism (CCM) is an important process that increases the local CO_2_ concentration near the active sites of the ribulose 1,5-bisphosphate carboxylase/oxygenase (RubisCO) enzyme in the carboxysome, a specialized subcellular organelle. During this process, inorganic carbon (Ci) in the form of HCO_3_^−^ is first transported by the Ci uptake systems encoded by *sbtA* (*slr1512*) and the *cmpABCD* operon (*slr0040-41* and *slr0043-44*) and then HCO_3_^−^ is converted to CO_2_ by carbonic anhydrase (*slr1347*) in the carboxysome. Both *sbtA* and *cmpC* were upregulated in the early light period (see Fig. S3A in the supplemental material; Fig. 2C). In addition, all other genes showed the global trend of having a very low expression level in the dark and a sharp increase at the start of the light period. Major structural genes for the carboxysome form the *ccmKLMN* operon, and temporal expression of all but one (i.e., *ccmK4*/*slr1839*) of these gene showed strong oscillatory behavior, with a peak at L3 and damping beyond that point in the light and all through the dark period (see Fig. S3A). Similarly, key genes for the Calvin cycle, such as RubisCO (*rbcSL*/*slr009* and *slr0012*), phosphoribulokinase (*prk*/*sll1525*), and glyceraldehyde-3-phosphate dehydrogenase (*gap2*/*sll1342*), showed behavior similar to that of the *ccmKLMN* operon, thus coupling the CCM to the Calvin cycle to ensure efficient CO_2_ fixation during the early light period (see Fig. S3B).

The transcriptional behavior of the glycolytic genes could be divided into three broad categories. The first includes genes encoding phosphoglucomutase (*pgm*/*sll0726*), glucose-6-phosphate isomerase (*pgi*/*slr1349*), phosphoglycerate kinase (*pgk*/*slr0394*), sedoheptulose-1,7-bisphosphatase (*glpX*/*slr2094*), triosephosphate isomerase (*tpiA*/*slr0783*), phosphoglycerate mutase (*gpm*/*slr1945*), and two isoforms of pyruvate dehydrogenase (*pdh*/*sll1721* and *slr1934*) that were upregulated at the beginning of the light period. The second category includes just one gene, encoding fructose-1,6-bisphosphatase (*fbp*/*slr0952*), that was upregulated in the entire dark period. Finally, the third category includes genes such as *pfkA*, *gap1*, *cbbA*, *fda*, *eno*, and *pyk* that did not show any strong cyclic behavior. Thus, except for *fbp*, the overall behavior of glycolytic genes indicates their strong coregulation with the genes from the Calvin cycle, the CCM, and photosynthetic machineries under L/D conditions (see Fig. S3C for details).

Cyanobacteria accumulate glycogen as a storage molecule in the light period and then use it as a carbon source in the dark period (29). In our study, gene for glycogen synthase (*glgC*, with two isoforms, *sll0945* and *sll1393*), a major gene in glycogen anabolism, showed cycling behavior, achieving the highest transcript level at the beginning of the light period. In contrast, for glycogen catabolism, one of the two isoforms of the essential gene for glycogen phosphorylase (*glgP*), namely, *slr1367*, showed sustained upregulation in the dark period (with a peak at D11), while the gene for isoamylase (*glgX*/*slr1857*) showed behavior similar to that of the *glgC* gene. This further suggests that glycogen metabolism might be under additional regulation, which is why *glgX* was upregulated in the light period. Overall, these data suggest that glycogen was accumulated in the early light period and consumed during the dark period (see Fig. S3D).

The OPPP plays a major role in the catabolism of glucose/glycogen (17) in *Synechocystis* PCC 6803 under mixotrophic or heterotrophic conditions and also interacts with the Calvin cycle under autotrophic conditions. Genes such as *tktA* (*sll1070*), encoding transketolase, *devB* (*sll1479*), encoding 6-phosphogluconolactonase, and *cfxE* (*sll0807*), encoding pentose-5-phosphate-3-epimerase, were upregulated in the earlier light period (with peaks at L1), while *rpiA* (*slr0194*), encoding ribose 5-phosphate isomerase, was upregulated in the later light period (i.e., L7). It is known that the roles of these genes are intertwined with the activities of the Calvin cycle, which is why their transcript levels are expected to be up in the light and down in the dark. Interestingly, the levels of the transcripts of all of these genes were damped down in the middle of the light period (see Fig. S4A in the supplemental material). On the other hand, none of the rest of the genes from the OPPP showed any cyclic behavior. Overall, the behavior of the genes involved in the Calvin cycle, glycolysis, and the OPPP is similar to that described in a recent study for another model cyanobacterium, *Synechococcus elongatus* strain PCC 7942 (hereafter, *Synechococcus* PCC 7942) (30). In the tricarboxylic acid (TCA) cycle, *glt* (*sll0401*, encoding citrate synthase) did not show any oscillatory behavior. However, the rest of the genes mostly had cycling behavior, even though sometimes with opposite phases. For instance, genes such as *icd* (*slr1289*), encoding isocitrate dehydrogenase, and *mdh* (*sll0891*), encoding malate dehydrogenase, were upregulated almost entirely during the dark period, with peaks at D1 and D11, respectively. In contrast, genes encoding fumarase (*fumC*/*slr0018*), succinyl coenzyme A synthetase (*sucD*/*sll1557*), malic enzyme (*me*/*slr0721*), and phosphoenolpyruvate carboxylase (*ppc*/*sll0920*) were upregulated in the early light period (i.e., L1 and L3) (see Fig. S4B). Similar to many other genes involved in central carbon metabolism, transcripts of these genes were damped down in the middle of the light period. On the basis of these data, a model could be proposed in which energy-producing (TCA cyclic) reactions are actively upregulated in the light period, whereas the production of major “hub metabolites” such as α-ketoglutarate and oxaloacetate are upregulated in the dark. However, further metabolomic studies are necessary to validate this model.

### (iv) Regulation.

KaiC is a hexameric protein that regulates the circadian rhythm by sequential phosphorylation and dephosphorylation at two distinct sites (31). Although the *kai* genes are believed to regulate circadian rhythms primarily at the phosphorylation level (32), the transcription of the *kai* genes in *Synechococcus* PCC 7942 is known to follow a circadian rhythm through negative feedback from KaiC (33). A similar phenomenon was also observed when the KaiABC oscillator system was reconstructed in the noncircadian bacterium *Escherichia coli* (34). In *Synechocystis* PCC 6803, transcripts of the members of the gene cluster orthologous to the well-studied *kai* genes in *Synechococcus* PCC 7942, namely, *kaiA* (*slr0756*), *kaiB1* (*slr0757*), and *kaiC1* (*slr0758*), peaked at the beginning of the light period, along with those of the orphan genes, namely, *kaiC3* (*slr1942*) and *kaiB2* (*sll1596*) (see Fig. S5A in the supplemental material) (35). However, only *kaiB2* showed a cycling transcript level. This observation can be correlated with the overall behavior of this organism, which has a low level of circadian rhythm (9). Two sigma factors, *sigI* (*sll0687*) and *sigC* (*sll0184*), were found to be upregulated in the entire dark period. While the function of *sigI* remains unknown, *sigC* plays a role in glycolysis, photosynthesis, and nitrogen metabolism (36).

Phytochromes are photoreceptors that detect light and trigger changes in gene expression. In *Synechocystis* PCC 6803, *cph1* (*slr0473*) is a phytochrome with a chromophore-bearing region at its N terminus and a histidine kinase consensus sequence at its C terminus, while *rcp1* (*slr0474*) is the cognate response regulator. It was previously reported that transcription of these two genes is repressed in the light and upregulated in the dark (37). Our data corroborated this expression pattern, showing that the transcripts of both genes peaked at D1 and reached a minimum in the early light period. However, only *rcp1* was found to be cyclic by our criteria (see Fig. S5A). Negative phototaxis is controlled by a two-component system composed of UirS/PixA (*slr1212*) and UirR/NixB (*slr1213*) (38). It has been suggested that UirR interacts with the promoter for a third gene, *lsiR*/*nixC* (*slr1214*), another response regulator. The transcription of *uirS*, *uirR*, and *lsiR* was cyclic, with downregulation in the light period (see Fig. S5A).

### (v) Metal transport.

Transition metals are essential biological micronutrients in photosynthetic organisms because of a high demand due to their involvement in the photosynthetic electron transport chain, as well as respiration. The transition metal ion requirement needs a transport network that regulates metal uptake, chelation, trafficking, and storage, as well as efflux export mechanisms (39). However, our current understanding of the principal features of the copper (Cu), iron (Fe), manganese (Mn), zinc (Zn), magnesium (Mg), cobalt (Co), molybdenum (Mb), and nickel (Ni) transport processes, as well as the mechanisms involved in their homeostasis within these autotrophic organisms, are not extensive.

Many of the transition metal transporters in *Synechocystis* PCC 6803 are known. These include the uptake systems for iron (Fut and Feo systems), the Mnt system specifying the ABC transporter-mediated delivery of manganese, the two-component copper response system CopRS (40) and the ZnuABC zinc uptake system (41). Export systems and efflux proteins for zinc, cobalt, copper, and nickel, as well as two P-type ATPase transporters for the delivery of copper to the cytoplasm and lumen, were also identified and analyzed (40, 42). Remarkably, the Fe, Zn, and Mn uptake systems were upregulated during the dark period, with a peak at D11, just before the onset of the light period (Fig. 2C). Moreover, *copS* (*sll0790*) is a transmembrane histidine kinase with a Cu^2+^ binding domain and a histidine kinase, and *copR* (*sll0789*) is the cognate response regulator. *copR* and *copS* exist as an operon along with *copM* (*sll0788*), a gene whose function is unknown. *copMRS* is induced by an increase in the copper concentration. Since CopR binds to the *copMRS* promoter, the operon may be subject to feedback regulation (40). All three of these genes were maximally expressed in the dark, with *copM* and *copR* being cyclic (with the highest expression at D11), indicating that the transcription of these genes may be under additional regulation. CopR also binds to the promoters for *copBAC* (*slr6042*, *slr6043*, and *slr6044*), a copper efflux system (40). However, the transcription of only *copC* showed cyclic oscillation in the opposite phase and with a lower amplitude, which might not be surprising, as the intracellular copper concentration was maximal in the light period.

### (vi) Translation and other cellular processes.

Of the 53 cyclic genes related to translation, 47 peaked either in the late dark period (D11) or in the early light period (i.e., L1/L3). The ribosomal protein cluster belongs to the latter category, whereas genes involved in protein translation (e.g., elongation factor P [*slr0434*], elongation factor EF-G [*sll1098*], and tRNA synthetase) and in protein breakdown (i.e., protease) are distributed across these two categories. On the basis of our data, the majority of the protein synthesis machinery was highly active in the early light period, whereas protein breakdown occurred in both the light and the dark periods (see Fig. S5B and C). The remaining cyclic genes (as represented in Table 1) in other categories, such as amino acid metabolism, biosynthesis of cofactors, and energy metabolism, showed behavior similar to that discussed so far. This further reiterates the enhanced transcriptional activity of *Synechocystis* PCC 6803 in the late dark and early light periods.

### Impact of diurnal rhythm on important metabolic pathways.

Barring a few exceptions, all of the genes associated with a specific cellular process exhibited similar temporal expression patterns. Here, we examined if the diurnal behavior of a particular cellular process was translated from the gene to the metabolic level. To this end, we made use of our previously developed genomic-scale model, *i*Syn731 (22). In total, 463 (out of 1,156) reactions showed cyclic behavior; 333 reactions were upregulated in the light period, while 130 reactions were upregulated in the dark. Specifically, we focused on the reactions involved in central metabolic pathways (i.e., the Calvin-Benson-Bassham [CBB] cycle/CCM, the pentose phosphate pathway, and the TCA cycle), glycogen synthesis/degradation, PSII/PSI, and transport processes.

**FIG 3:**
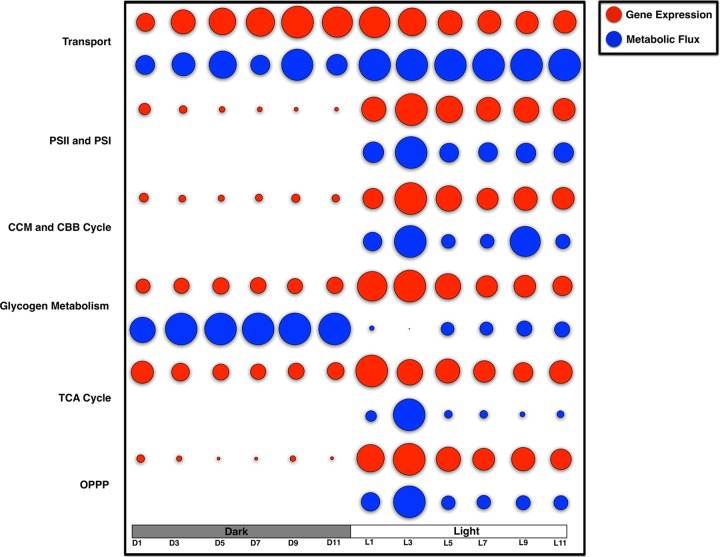
Relative changes in the expression levels (A) and metabolic fluxes (B) of major metabolic genes over the L/D cycle. Here, both gene expression and metabolic flux were scaled between 0 and 1.

Figure 3 represents the relative changes in the levels of gene expression and metabolic flux of each of these cellular processes. By incorporating cyclic gene expression as the regulatory constraint, the model-predicted flux changes, in general, were well correlated with the levels of gene expression (see Materials and Methods for details). However, glycogen metabolism was one notable exception. In the dark phase, the model-predicted flux was relatively higher, whereas the gene expression showed otherwise. Since maximization of the dark-phase biomass was set as an objective function by utilizing glycogen (as the sole carbon source), a higher flux of glycogen metabolism was not unusual. In addition, the model-predicted fluxes of PSII/PSI, the CBB cycle/CCM, the TCA cycle, and the OPPP during the dark period were rather low. This further implies the presence of complex regulatory mechanisms that are beyond the scope of such a metabolic model. Although several other factors, such as protein formation/folding/activation and posttranslational modifications, can play important roles in determining the metabolic/cellular behavior of any biological system, given the scope of this work, the model made the simple assumption that gene expression is linearly correlated with metabolic flux and predicted the relative changes in the flux level over the L/D cycle quite well.

### Cycling of physiological parameters.

In order to detect the change in physiology corresponding to the transcriptional behavior in the L/D cycle, samples were collected in two diurnal cycles to test the physiological parameters of the cells. We found that cell density (optical density at 730 nm) increased only during the light period (data not shown), which was consistent with what was previously reported (43). In addition, measurements of oxygen production and consumption rates (see Fig. S6 in the supplemental material for details), glycogen concentrations, ATP levels, and NADPH/NADP^+^ ratios during the entire diurnal process revealed interesting L/D variations in *Synechocystis* PCC 6803 cells.

### Glycogen concentration, total ATP, and O_2_ production and consumption rates.

**FIG 4:**
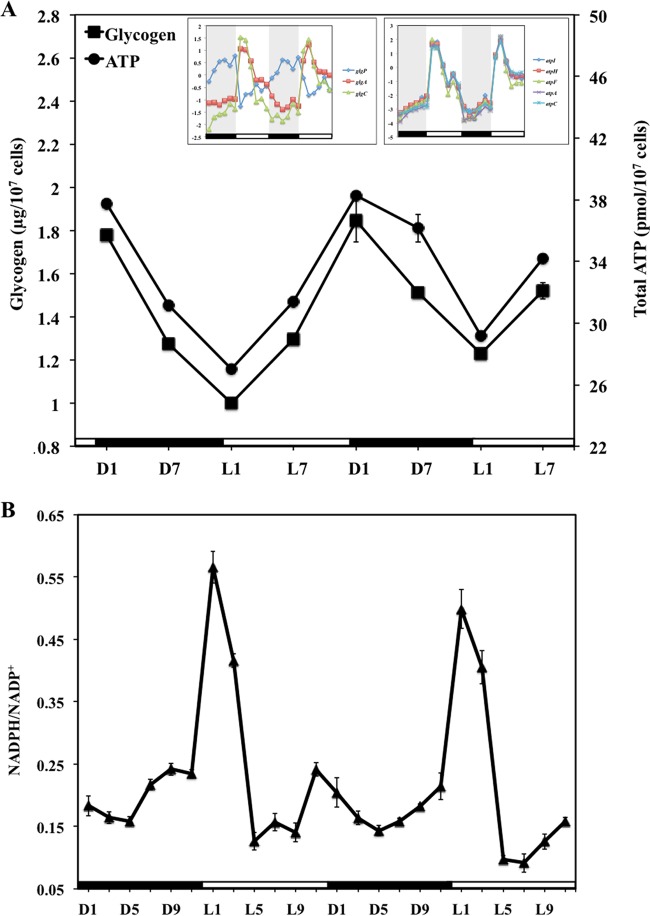
Glycogen and ATP levels (A) and NADPH/NADP^+^ ratios (B) in cells during diurnal cycles. The transcriptional oscillation of genes involved in glycogen metabolism and ATP synthesis is shown in the insets in panel A. Cells were autotrophically cultured in BG11 medium under alternating 12-h L/D cycle conditions. Data points are the mean ± the standard deviation of three biological replicates.

Glycogen is the main carbon storage in cyanobacteria (44). Our measurements showed that it began to accumulate during the light period and, as expected, it was degraded in the dark. The total ATP level also showed a cycling pattern similar to that of glycogen (Fig. 4A). Thus, these results were correlated with the temporal changes in the genes involved in glycogen metabolism and ATP synthesis (as discussed earlier). Furthermore, cells in the dark could be inferred to behave similarly to those under heterotrophic conditions, which basically led us to the measurement of O_2_ production and consumption rates. Samples from the light period were tested with NaHCO_3_ as the electron acceptor, while the oxygen consumption rate was assayed in the absence of any light. The results (see Fig. S6 for details) showed that O_2_ was produced at similar rates during the light period, whereas it was consumed during the dark period because of respiratory activity. The variations in glycogen, total ATP, and O_2_ during the L/D cycle indicated differences in *Synechocystis* PCC 6803 metabolism in the presence and absence of light.

### Cellular NADP(H) level.

In order to explore diurnal variations in the reducing equivalent levels in *Synechocystis* PCC 6803 cells, the NADPH/NADP^+^ ratio was measured over two consecutive L/D cycles (similar to the microarray experiment). On the basis of our measurements, the NADPH/NADP^+^ ratio (Fig. 4B) oscillated with time, however in a different way than the total ATP and glycogen concentrations (Fig. 4A). Cellular ATP and glycogen concentrations started decreasing at the beginning of the dark period and increased continuously when light was available. In contrast, the NADPH/NADP^+^ ratio was highest at the transition from dark to light. It then decreased at L3 and ultimately reached the lowest level at L5. The ratio did not change much beyond L5 and all through the dark period until the next light phase started. The changing pattern of the ratio is similar to that of the genes from light harvesting/photosynthesis (see Fig. S1A and B in the supplemental material). Therefore, this ratio has a fast response to transcriptional variation and also is tightly coregulated with the photosynthetic genes.

## DISCUSSION

Diurnal oscillation in cyanobacteria has been the focus of many studies (9, 29). However, little is known about how the transcriptional changes in *Synechocystis* PCC 6803 affect its metabolic and physiological characteristics during the diurnal cycle (9). The present study was focused on understanding the impact of diurnal changes on the physiology of this model cyanobacterium by analyzing the changes in the global transcriptional level, *in silico* metabolic fluxes, and important physiological parameters.

Our gene expression measurements and subsequent analysis (as explained in Materials and Methods) revealed that 39% of the *Synechocystis* PCC 6803 genes show cycling behavior in two alternate L/D cycles. Two separate gene coregulation networks (i.e., one whose expression peaks in the light and one whose expression peaks in the dark), as developed on the basis of this data set, further revealed how genes involved in diverse metabolic processes were coregulated in anticipation of the presence or absence of light (see Movie S1 in the supplemental material). Hence, this implies that strong transcriptional regulation exists among various cellular processes. Extending previous studies that showed strong coregulation of either neighboring (45) or functionally associated genes (29), our work reveals a unique feature of *Synechocystis* PCC 6803 physiology in that the majority of cellular processes are active during the light period, leading to the development of a transcriptional network whose expression peaks in the light, and only a few other cellular processes (e.g., metal transport, maintenance, and regulation) occurred mostly in the dark, as represented by a transcriptional network whose expression peaks in the dark.

Our temporal-expression-based study also suggested that phycobilisomes are degraded during the late dark period and then resynthesized mainly in the early light period. While the temporal profiles of the CCM, the Calvin cycle, photosynthetic electron transport, ATP synthesis, and glycogen metabolism were as expected, the genes involved in respiration, such as those for NADH dehydrogenase and cytochrome *c* oxidase, showed expression profiles analogous to those of the genes involved in photosynthesis. In this context, it is noteworthy that photosynthesis and respiration are both localized to the thylakoid membrane and also have some electron carriers in common (46). TCA cycle genes associated with energy production were upregulated in the light period. In contrast, genes involved in the production of major “hub metabolites” such as α-ketoglutarate and oxaloacetate were upregulated in the dark.

In addition, most of the protein synthesis machinery was highly active in the early light period. Most interestingly, systems that take up transition metals, key players in photosynthetic and respiratory electron transport chains, were up in the late dark period and damped down in the middle of the light period. These findings suggest that *Synechocystis* PCC 6803 sequesters necessary metals and performs optimal regulation (by minimizing energy-consuming metabolic activities) in the dark so that it can synthesize proteins, generate energy, and carry out carbon fixation in the ensuing light period. Herein, another key point is the times when the genes whose expression peaks in the light and those whose expression peaks in the dark were maximally expressed. While the behavior of the majority of the genes whose expression peaks in the dark are correlated with the “anticipation of change” in the following hour, it is not clear why *Synechocystis* PCC 6803 downregulates the majority of the genes whose expression peaks in the light beyond L5, when resources such as light and CO_2_ are plentiful. Had the physiology of *Synechocystis* PCC 6803 been driven only by the presence or absence of light, photosynthesis and other related processes would be upregulated with the onset of light and their transcript levels would substantially be kept higher over the entire light period. Hence, the diurnal processes in *Synechocystis* PCC 6803 seem to be driven by both light and an endogenous regulatory system that highly activates certain genes at the end of the dark period so that certain others achieve maximal transcript levels at the early stage of the light period. In addition, as soon as the aggregate cellular requirements are met, transcript levels of these genes are ramped down during the latter half of the light period.

In order to explore if the diurnal behaviors of transcripts were translated to the physiological and metabolic levels, we predicted fluxes by utilizing our previously developed genomic-scale metabolic model, *i*Syn731. The relative flux changes in the major cellular processes (such as the CBB cycle/CCM, the OPPP, and the TCA cycle) were well correlated with the relative changes in the associated gene expression levels. Therefore, just by using metabolic and regulatory information (via gene expression levels), the model showed how the aggregate metabolic behavior of major cellular pathways was dictated by the diurnal lifestyle of *Synechocystis* PCC 6803*.* In addition, we measured important physiological parameters, including glycogen concentrations, total ATP levels, oxygen production and consumption rates, and NADP(H) levels. During the early light period, photosystem, RubisCO, and glycogen synthesis genes were upregulated, while during the late dark period, glycogen degradation genes were upregulated. Consistent with these findings, cellular glycogen levels decreased in the dark period and eventually reached a minimum level around the dark-to-light transition time. However, contrary to the overall behavior of the glycogen synthesis and other photosynthetic genes that reached their peak expression levels at L1/L3/L5 and were damped down beyond then, glycogen levels continuously increased during the light period to reach a maximum at the light-to-dark transition time. Therefore, our inference is that glycogen synthesis machinery might require some kind of “priming” at the beginning of the light period that is served by the highest expression levels of the relevant genes. Beyond that time point, it can maintain the level of glycogen production even with lower gene expression levels. A similar inference can also be drawn for the ATP level going continuously up and down during the light and dark periods, respectively. In addition to the downregulation of the genes for ATP synthase over the dark period, upregulation of energy-intensive processes such as transport and regulation (which peaked at D11) presumably plays a significant role in the decrease in total ATP, reaching a minimum level at the dark-to-light transition.

**FIG 5:**
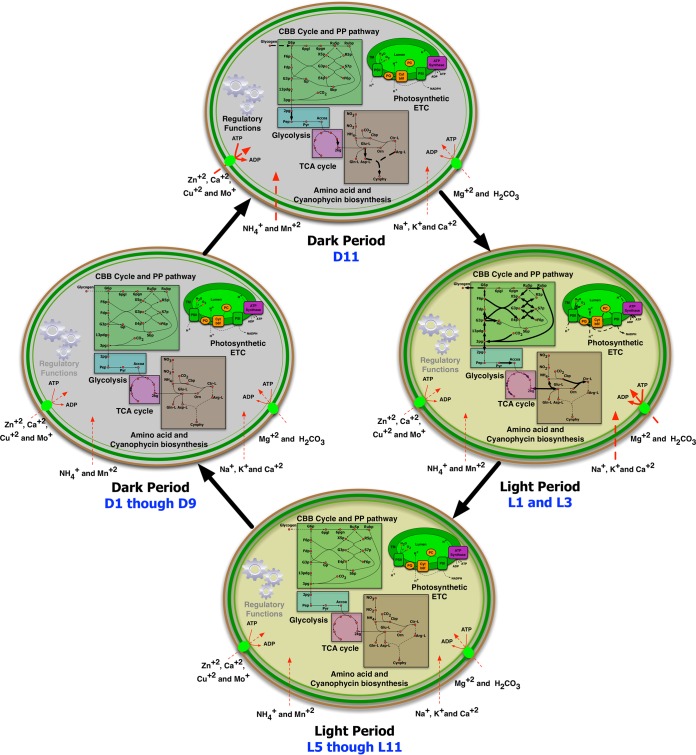
Schematic representations of the differences in *Synechocystis* PCC 6803 during light and dark periods. Four phases of the diurnal cycles are highlighted: D1 through D9, D11, L1/L3, and L5 to L11. The thicknesses of the arrows in the metabolic map are proportional to the activities of specific metabolic or transport reactions under given conditions. Gray and black regulatory functions represent inactive and active regulatory genes, respectively. G6P, glucose 6-phosphate; Ru5p, ribulose 5-phosphate; Rubp, ribulose-1,5-bisphosphate; 6pgl, 6-phosphogluconolactone; 6pgn, 6-phosphogluconate; F6p, fructose 6-phosphate; X5p, xylulose 5-phosphate; R5p, ribose 5-phosphate; Fdp, fructose 1,6-bisphosphate; G3p, glyceraldehyde 3-phosphate; S7p, sedoheptulose 7-phosphate; Gp, glycerone phosphate; E4p, erythrose 4-phosphate; 13pdg, 1,3-bisphospho-d-glycerate; 3pg, 3-phosphoglyceric acid; Sbp, sedoheptulose-1,7-bisphosphate; Pep, phosphoenolpyruvate; Pyr, pyruvate; Accoa, acetyl CoA-carboxylase; 2kg, 2-ketoglutarate; Cbp, carbamoyl phosphate; Cynphy, cyanophycin; TM, transmembrane; ETC, electron transport chain; PC, plastocyanin; PQ, plastoquinone; Cyt b6f, cytochrome b6f complex.

The NADPH/NADP^+^ ratio varied more than the total ATP level during the L/D cycle. During the early light period, a sufficient amount of NADPH, a necessary cofactor for growth, was produced mainly by a photosynthetic electron transport chain, whereas in the dark period, it was generated in a lesser amount via central carbon metabolism. Thus, changes in the NADPH/NADP^+^ ratio were well correlated with the transcriptomic behavior of genes for photosynthesis during the L/D cycle, suggesting that this ratio is an endogenous regulator that plays a major role in the diurnal entrainment of *Synechocystis* PCC 6803. As reported elsewhere (14, 47), other factors, in addition to environmental time cues, that regulate circadian behavior include NAD^+^-dependent deacetylases SIRT1 and SIRT6 (for mammals) and the metabolic homeostasis (e.g., ATP/ADP) ratio (for the cyanobacterium *Synechococcus* PCC 7942). Therefore, the feedback signal from NADPH/NADP^+^ can be the endogenous regulator that plays a major role in diurnal entrainment in *Synechocystis* PCC 6803. On the basis of both transcriptomic and physiological analyses, Fig. 5 represents important cellular processes in *Synechocystis* PCC 6803 and their corresponding regulation in four different phases, namely, D1 to D9, D11, L1/L3, and L5 to L11. This further emphasizes that the majority of the physiological transitions occur at D11 and L1/L3 under L/D conditions (see Movie S2 in the supplemental material for details).

Overall, the present study provides valuable insights into the temporal changes in the physiology of *Synechocystis* PCC 6803 during the diurnal cycle. Compared with our earlier observations on the unicellular diazotrophic cyanobacterium *Cyanothece* sp. strain ATCC 51142 (hereafter, *Cyanothece* ATCC 51142) (29), these results highlight important differences in this nondiazotrophic cyanobacterium. In *Cyanothece* ATCC 51142, a number of cellular processes, such as glycolysis, the OPPP, amino acid biosynthesis, the TCA cycle, nitrogen fixation, and cyanophycin biosynthesis, are upregulated mostly during the dark period, while in *Synechocystis* PCC 6803, only cyanophycin biosynthesis is upregulated in the dark. In addition, a large number of genes of *Cyanothece* ATCC 51142 have maximal transcript levels during the transitions from light to dark and from dark to light, in contrast to genes whose expression peaks only during the dark-to-light transition in *Synechocystis* PCC 6803. Evidently, given the absence of the energy-intensive and oxygen-sensitive nitrogen fixation process, *Synechocystis* PCC 6803 does not require the complex regulation patterns that *Cyanothece* ATCC 51142 does. Instead, *Synechocystis* PCC 6803 uses the simple rule of minimizing cellular processes in the dark, thus preserving energy, and tuning its physiology at the dark-to-light transition to make the best use of light at the onset of a new day.

In this study, we focused on the diurnal variations in *Synechocystis* PCC 6803 at the gene expression, metabolic flux, and physiological levels. Even though *Synechocystis* PCC 6803 is used as a model strain in synthetic-biology studies (48), its full potential has yet to be explored by exploiting its diurnal lifestyle. In addition, one of the major advantages of developing *Synechocystis* PCC 6803 as a bioproduction platform is to utilize solar energy; which is why it is critical to explore the L/D variations in its cellular physiology. Hence, this work provides a basis for understanding not only the impact of the diurnal cycle on the physiology of *Synechocystis* PCC 6803 but also the differences between diazotrophic and nondiazotrophic cyanobacteria, which may lead to exciting metabolic engineering/synthetic-biology applications such as incorporating nitrogen fixation ability in *Synechocystis* PCC 6803.

## MATERIALS AND METHODS

### Strain and culture conditions.

Wild-type *Synechocystis* PCC 6803 was photoautotrophically cultured in BG11 medium (49) in shaking flasks at 30°C under continuous illumination with 50 µmol of photons of white light m^−2^ s^−1^. For culturing and sampling of cells in alternating 12-h L/D cycles, *Synechocystis* PCC 6803 cells from continuous-light conditions were diluted 200-fold and cultured under L/D cyclic conditions at the same temperature and light intensity but bubbled with air, which was set up as the preculture. After 7 days of acclimation to the cyclic conditions, cells were diluted again 100-fold and after 4 days, cells were sampled every 2 h for 48 h, starting with 1 h into the dark period (time point D1). A total of 24 samples were collected.

### Microarray and data analysis.

The microarray we designed in the same way as in the past (50). RNA isolation and microarray experiments were done as in our previous study (29). An equimolar mixture of RNA samples from all time points was used as the control.

In order to correct for variations in labeling intensity between channels, data from each microarray were normalized independently. For each measurement, the background signal was subtracted from the raw signal. Then, for individual probe, a locally weighted scatterplot smoothing (LOWESS) curve was fitted to the training set by using a window length of 25% of the total data points. Subsequently, the signals were normalized with respect to the fitted LOWESS curve via linear interpolation in order to correct points falling between points in the training set. Finally, after LOWESS normalization, log_2_ (experiment/control) ratios were computed for each probe (29). For genes having multiple probes, average log_2_ ratios were calculated as gene expression levels at specific time points. Since the ranges of log_2_ ratios differ significantly across the genes, genes with broad ranges govern the identification of cyclic genes based on any log_2_ cutoff or amplitude/period of the model based on the log_2_ ratios. Therefore, we applied feature scaling by normalizing log_2_ ratios in a range of +1 to −1 so that each gene expression profile contributed equally to the cyclic gene decision-making process.

Similar to the method used by Kucho et al. (10), we used a modified cosiner method to analyze the rhythmicity of temporal expression patterns. Here, *g_1_*, *g_2_*, *… g*_24_ are the feature-scaled log_2_ ratios at time points *t_1_*, *t_2_*, *… t_24_* for an individual gene. After performing linear regression analysis with the data set by the least-squares method, we derived a regression line for each gene as follows:

f(t)=αt+β

where *f*(*t*) is the linear trend of the temporal expression data. In order to prevent incorrect cosine curve fitting especially for those genes having nonzero α or β values, we detrended data as follows:

$$g_{i}\prime =g_{i}-f(t_{i})$$

and subsequently fitted the detrended data to 3,482 cosine curves (corresponding to 3,482 genes) of *Fj*(*t*) (where, *j* = 1 to 3,482) with a series of period lengths (*T_j_*) of 12 to 36 h at 0.1-h intervals by the Fourier transformation method, as represented in the following equations:

$$F_{j}(t)=\sqrt{a^{2}+b^{2}}\;\cos\;[(2\pi t/T_{j})-\varphi ]$$

where

$$a=\left(2/n\right)\overset{n}{\underset{i=1}{\sum }}\,\;g_{i}\prime \;\cos\left(2\pi t_{i}/T_{j}\right)$$

$$b=(2/n)\overset{n}{\underset{i=1}{\sum }}{\;g}_{i}\prime \;\sin(2\pi t_{i}/T_{j})$$

$$\text{Acrophase},\varphi ={\text{tan}}^{-\text{1}}(b\text{/}a)$$

Here, *n* is set to 24. To determine the level of the fit, we calculated the error factor in the following manner:

$$Ef=\frac{\sqrt{\overset{n}{\underset{i=1}{\sum }}{\left[g_{i}\prime -F_{j}(t_{i})\right]}^{2}}}{n}$$

Then, the best-fit cosine curve for each gene was chosen as *F_j_*(*t*) with a minimum *Ef* value and the amplitude and peak expression time were calculated as follows:

$$\text{Amplitude}=2^{\sqrt{a^{2}+b^{2}}}$$

$$\mathrm{Peak}\;\mathrm{expression}\;\mathrm{time}\;\mathrm{(in}\;\mathrm{circadian}\;\mathrm{time})=\frac{24\varphi }{2\text{π}}$$

The set of cyclic genes was identified on the basis of three criteria in a hierarchical manner (10). For instance, a gene shows cyclic behavior if (i) its period is between 18 and 26.8 h, (ii) its *Ef* is ≤0.2, and (iii) its *P* value is <0.05 in a Student *t* test performed to differentiate expression levels at peak and trough time points and false positives are controlled by applying Holm’s method.

To develop a coexpression network, Pearson correlations were calculated between all gene pairs by using their feature-scaled log_2_ ratios. Gene pairs having Pearson coefficients of ≥0.90 were then connected (29). Cytoscape (23, 24) version 3.1.1 was used to visualize the network. Cyclic metabolic genes involved in our previously developed *Synechocystis* PCC 6803 genomic-scale model (*i*Syn773) (22) were mapped to reactions via gene-protein-reaction (GPR) associations. To this end, the underlying assumption was that any reaction involving at least one cyclic gene was considered to show cyclic behavior.

### Identification of cyclic reactions from the *i*Syn731 model, mapping of cyclic gene expression, and calculation of fluxes through major pathways.

Cyclic reactions were identified from the *i*Syn731 model on the basis of GPR associations. Any reaction was identified as cyclic if at least one of the associated genes was cyclic. Cyclic gene expression were mapped to the genomic-scale *Synechocystis* PCC 6803 model (*i*Syn731) as regulation of reaction fluxes similar to that described elsewhere (51). The flux distribution at each of the time points (in the dark and light periods) was inferred by using FBA (21) as follows:

$$\text{Maximize}{\;v}_{\text{Biomass}{}_{\mathrm{diurnal}\text{ condition}}}\forall \mathrm{diurnal}\;\mathrm{condition}\;\in \;\mathrm{Dark,}\text{ Light}$$

subject to

$$\overset{m}{\underset{j=1}{\sum }}s_{ij}v_{j}=0\;\forall \;i\;\in \;1,\;\ldots \;,n$$

$$a_{j}v_{j}^{\min}\leq v_{j}\leq a_{j}v_{j}^{\max}\;\forall \;j\;\in \;1,\;\ldots \ldots \;,m$$

$$\;0\leq v_{\text{Nutrients}}\leq v_{\text{Nutrients}}^{\text{max}}\;\forall \;\mathrm{Nutrients}\in \mathrm{Light,}\mathrm{Carbon}\text{ source(s),}\mathrm{Micronutrients}$$

Here, *S*_ij_ is the stoichiometric coefficient of metabolite *i* in reaction *j* and *v_j_* is the flux value of reaction *j*. The parameters $v_{j}^{\min}$ and $v_{j}^{\max}$ are the minimum and maximum allowable fluxes for reaction *j*, respectively. Parameter *a_j_* is the scaling factor based on gene expression. The expression level of each cyclic gene was scaled between 0 and 1. Since expression levels were measured in two consecutive diurnal cycles, the expression of any cyclic gene at a specific time point was then calculated as an average of these two consecutive measurements. Note that on the basis of the cyclic behavior of genes, reactions of the model *i*Syn731 were categorized into two groups: cyclic reactions and noncyclic reactions. For each cyclic reaction catalyzed by a protein complex, *a_j_* was set to the minimum expression levels of the cyclic genes involved. In addition, for a cyclic reaction catalyzed by isozymes, *a_j_* was set to the sum of the expression levels of the associated cyclic genes. For all of the remaining noncyclic reactions, *a_j_* was set as 1. *v_Biomass_* represents two distinct biomass equations in the light and dark phases. *v_Nutrient_* represents the uptake reactions of the necessary nutrients such as carbon sources (glycogen and CO_2_/H_2_CO_3_ in the dark and light phases, respectively), light, and micronutrients. Therefore, the flux distribution at a particular time point was calculated by setting an appropriate biomass equation/growth condition (light versus dark) and also incorporating cyclic gene expression as a regulatory constraint on relevant reactions. Flux values through major pathways (i.e., the CCM and CBB cycles, the OPPP, the TCA cycle, PSII/PSI, glycogen metabolism, and transport processes) at specific time points were calculated by averaging the fluxes of the reactions involved in specific pathways/subsystems. Similar to cyclic gene expression, these fluxes (of individual pathways) were then scaled between 0 and 1.

CPLEX solver (version 12.4, IBM ILOG) was used in the GAMS (version 24.4.4; GAMS Development Corporation) environment to solve the aforementioned optimization models. All computations were carried out on Intel Xeon E5450 Quad-Core 3.0 GHz and Intel Xeon X5675 Six-Core 3.06 GH, which are part of the lionxj and lionxf clusters (Intel Xeon E- and X-type processors and 128 and 128 GB of memory, respectively) of the High Performance Computing Group of The Pennsylvania State University.

### Whole-cell absorbance.

Absorption spectra of whole-cell samples at eight time points (D1, D7, L1, and L7 for two cycles) between 400 and 750 nm were recorded on a DW2000 spectrophotometer and normalized to the optical density at 730 nm.

### Determination of oxygen production and consumption rates.

Oxygen production and consumption rates of cell suspension were measured with a Clark-type electrode (45). Cells were resuspended in fresh BG11 medium to a chlorophyll concentration of 5 µg/ml and incubated at 30°C. Oxygen production assays were performed with BG11 medium with 10 mM sodium bicarbonate as the electron acceptor. Light was provided via a fiber-optic light guide with neutral gray filters for tuning the light intensity to 230 µmol of photons m^−2^ s^−1^, which was chosen for the maximum oxygen production rate without photodamage. To measure respiration, the cell suspension was kept in the dark to test oxygen consumption. Data are presented as the mean ± the standard deviation of three biological and two technical replicates.

### Determination of glycogen, total ATP, and NADP(H) levels.

Glycogen levels were assayed by the method developed by Osanai et al. (52). Briefly, 1 ml of *Synechocystis* PCC 6803 cells was suspended in 100 µl of 3.5% (vol/vol) sulfuric acid and boiled for 40 min. The glucose in the hydrolysate was assayed by *o*-toluidine solution, and absorbance at 630 nm was subsequently measured.

The total ATP concentration in cells was determined by using an ATP fluorometric assay kit (BioVision, CA) according to the manufacturer’s instruction. Cells were lysed in the buffer supplied in the kit, and the product generated by the reaction with ATP was quantified by measuring fluorescence intensity (excitation and emission wavelengths of 535 and 587 nm, respectively) with a fluorometric plate reader (BioTek Instruments, Winooski, VT).

Both NADP^+^ and NADPH levels in cells were assayed with an NADP/NADPH quantification kit (Sigma-Aldrich, MO), which is specific for NADP^+^ and NADPH. Briefly, the total NADP (NADP^+^ and NADPH) was extracted and samples were heated at 60°C for 30 min, cooled on ice, and centrifuged to leave only NADPH. Total-NADP and NADPH-only samples were individually quantified with a colorimetric assay by using absorbance at 450 nm on a plate reader.

### Microarray data accession number.

The microarray data, including complete information on the microarray design, are accessible in the GEO database under accession number GSE79714.

## SUPPLEMENTAL MATERIAL

Data Set S1  Distribution of 1,345 cyclic genes across different pathways/subsystems and their corresponding gene expression patterns. Download Data Set S1, XLSX file, 3.6 MB

Figure S1  Expression profiles of genes with cyclic patterns that are involved in light harvesting (A), ATP synthesis (B), and the *cytb_6_f* complex (C). L/D cycles are indicated as gray and white bars below the *x* axis, respectively. The log_2_ ratios of transcript abundance to the pooled sample control are plotted on the *y* axis. Download Figure S1, TIF file, 2.6 MB

Figure S2  Expression profiles of genes with cyclic patterns that are involved in the photosynthetic electron transport chain (A), NADH dehydrogenase (NDH) (B), and cytochrome *c* oxidase (C). L/D cycles are indicated as gray and white bars below the *x* axis, respectively. The log_2_ ratios of transcript abundance to the pooled sample control are plotted on the *y* axis. Download Figure S2, TIF file, 2.6 MB

Figure S3  Expression profiles of genes with cyclic patterns that are involved in the CCM (A), the Calvin cycle (B), glycolysis (C), and glycogen metabolism (D). L/D cycles are indicated as gray and white bars below the *x* axis, respectively. The log_2_ ratios of transcript abundance to the pooled sample control are plotted on the *y* axis. Download Figure S3, TIF file, 3 MB

Figure S4  Expression profiles of genes with cyclic patterns that are involved in the OPPP (A) and the TCA cycle (B). L/D cycles are indicated as gray and white bars below the *x* axis, respectively. The log_2_ ratios of transcript abundance to the pooled sample control are plotted on the *y* axis. Download Figure S4, TIF file, 1.4 MB

Figure S5  Expression profiles of genes with cyclic patterns that are involved in regulation (A), genes with maximal expression in the dark that are involved in translation (B), and genes with maximal expression in the light that are involved in translation (C). L/D cycles are indicated as gray and white bars below the *x* axis, respectively. The log_2_ ratios of transcript abundance to the pooled sample control are plotted on the *y* axis. Download Figure S5, TIF file, 2.6 MB

Figure S6  Oxygen production and consumption of *Synechocystis*. Samples collected at dark time points were always kept without any light to test respiration in the dark. Negative values represent oxygen consumption. Samples collected at light time points were tested with NaHCO_3_ as the electron acceptor to measure the oxygen production rate. Data are presented as the mean ± the standard deviation of three biological replicates. Download Figure S6, TIF file, 1.4 MB

Movie S1  Coexpression network created with the Cytoscape program showing two disjoint networks: one that contains 781 genes whose expression peaks in the light period and the other with 522 genes whose expression peaks in the dark. Download Movie S1, MOV file, 4.5 MB

Movie S2  Video showing the physiological transitions occurring in the light/dark conditions especially in the late dark (i.e., D11) and early light (L1/L3) phases. Download Movie S2, MOV file, 1.2 MB
