# Supplementary figures and images for: Diurnal Regulation of Cellular Processes in the Cyanobacterium Synechocystis sp. Strain PCC 6803: Insights from Transcriptomic, Fluxomic, and Physiological Analyses
Source: mBio. 2016 May 3;7(3):e00464-16. doi: 10.1128/mBio.00464-16 (PMC4959675; doi:10.1128/mBio.00464-16)

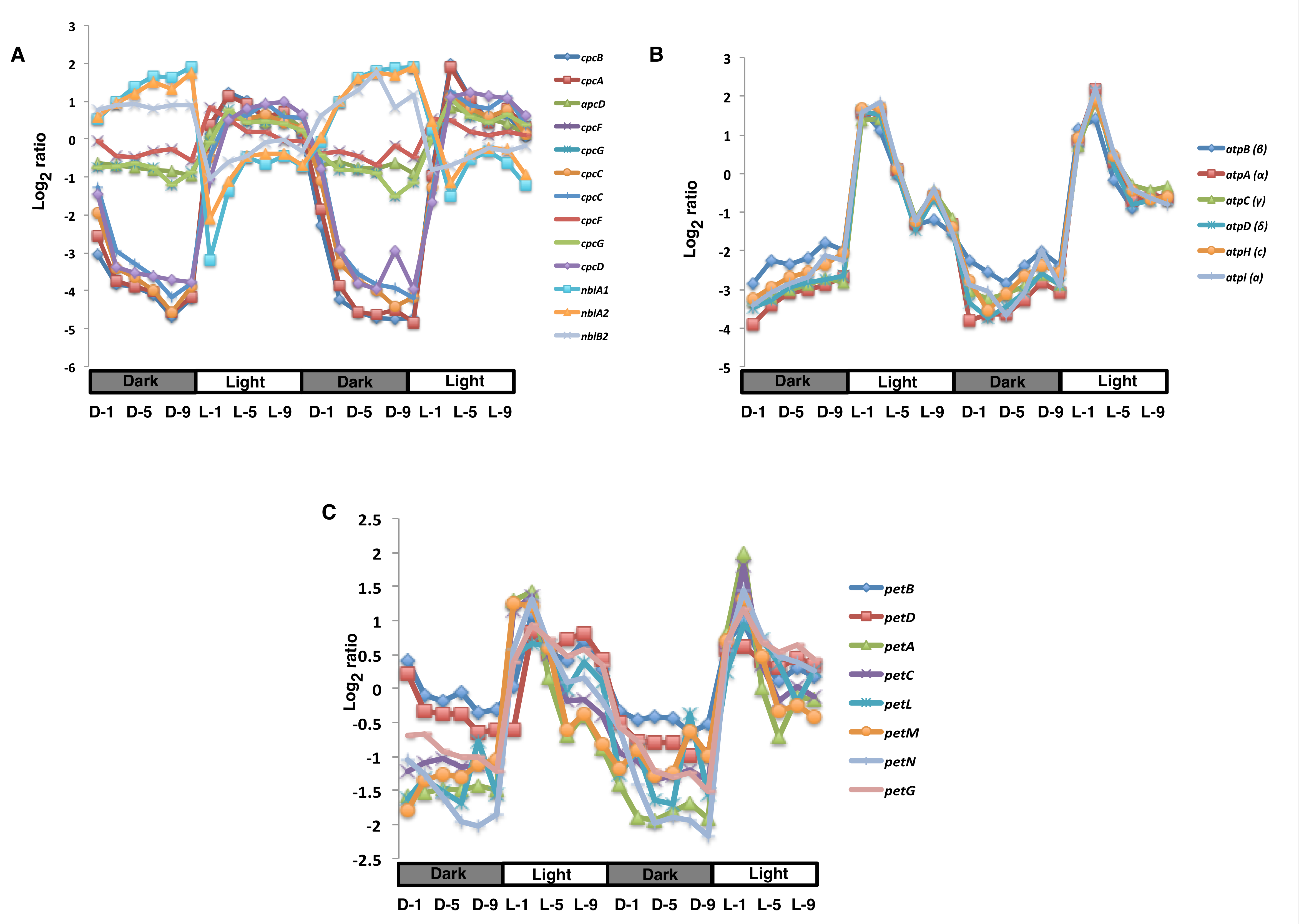

Supplement: Figure S1 — Expression profiles of genes with cyclic patterns that are involved in light harvesting (A), ATP synthesis (B), and the cytb6f complex (C). L/D cycles are indicated as gray and white bars below the x axis, respectively. The log2 ratios of transcript abundance to the pooled sample control are plotted on the y axis. Download [file mbo002162797sf1.tif]

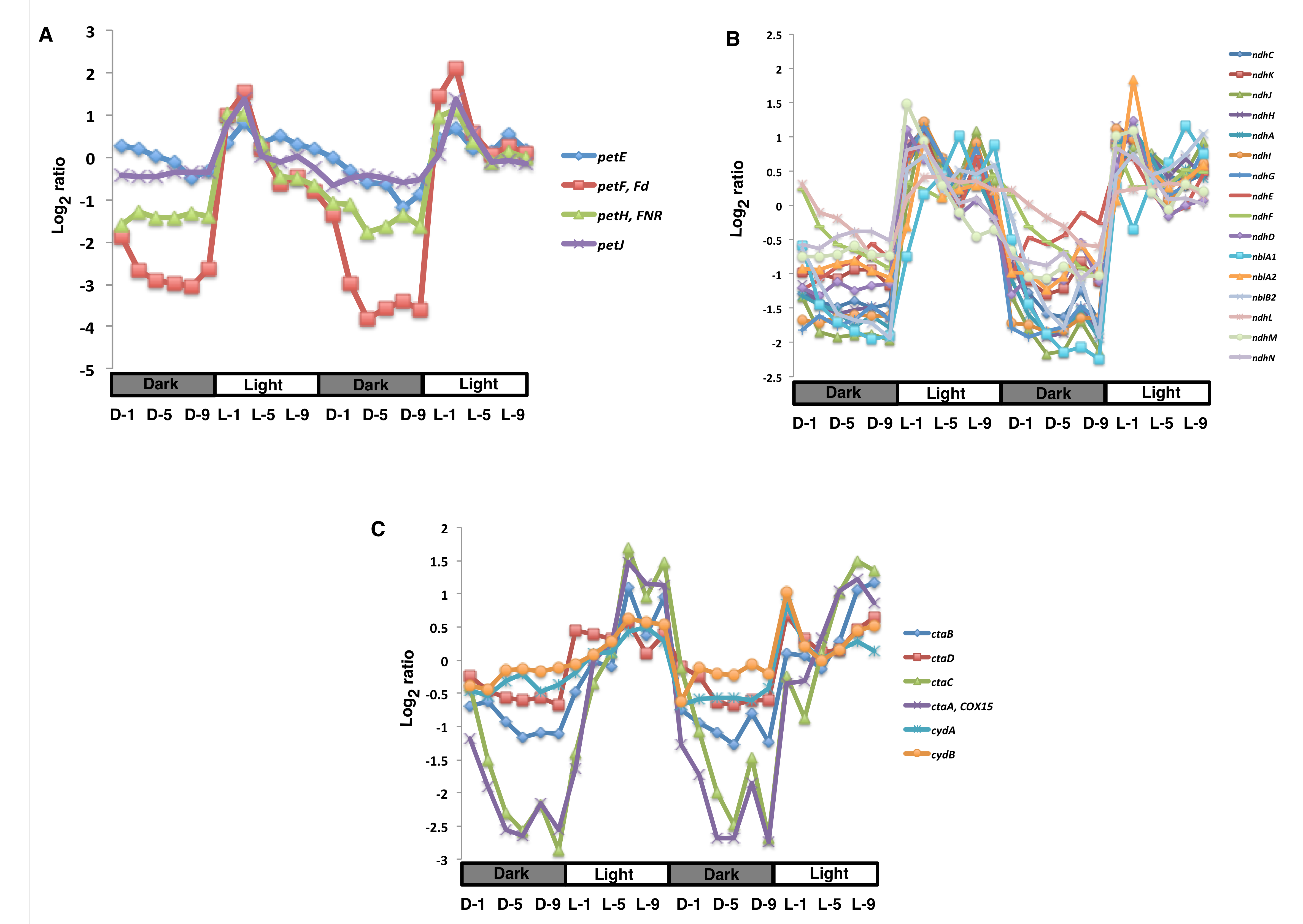

Supplement: Figure S2 — Expression profiles of genes with cyclic patterns that are involved in the photosynthetic electron transport chain (A), NADH dehydrogenase (NDH) (B), and cytochrome c oxidase (C). L/D cycles are indicated as gray and white bars below the x axis, respectively. The log2 ratios of transcript abundance to the pooled sample control are plotted on the y axis. Download [file mbo002162797sf2.tif]

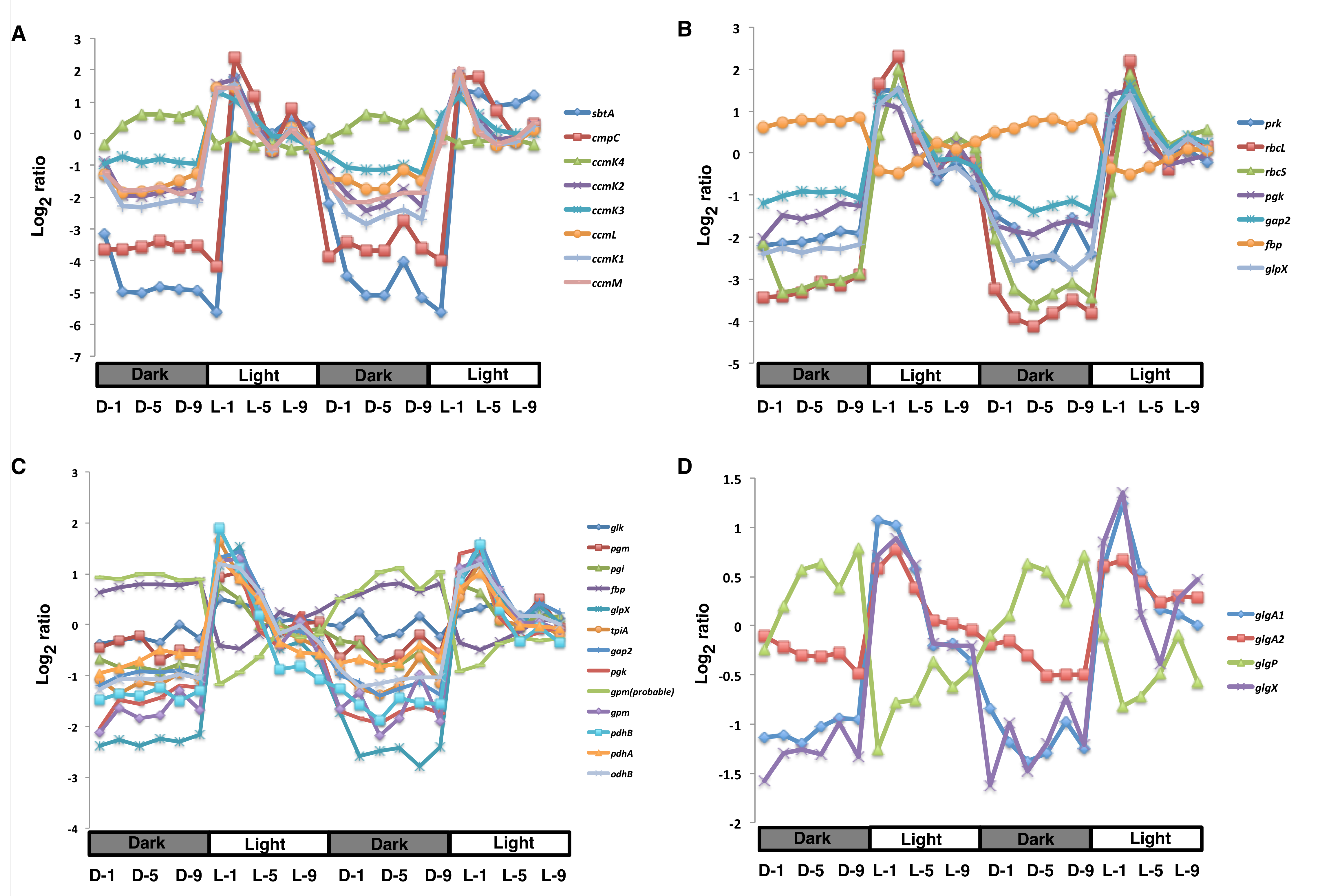

Supplement: Figure S3 — Expression profiles of genes with cyclic patterns that are involved in the CCM (A), the Calvin cycle (B), glycolysis (C), and glycogen metabolism (D). L/D cycles are indicated as gray and white bars below the x axis, respectively. The log2 ratios of transcript abundance to the pooled sample control are plotted on the y axis. Download [file mbo002162797sf3.tif]

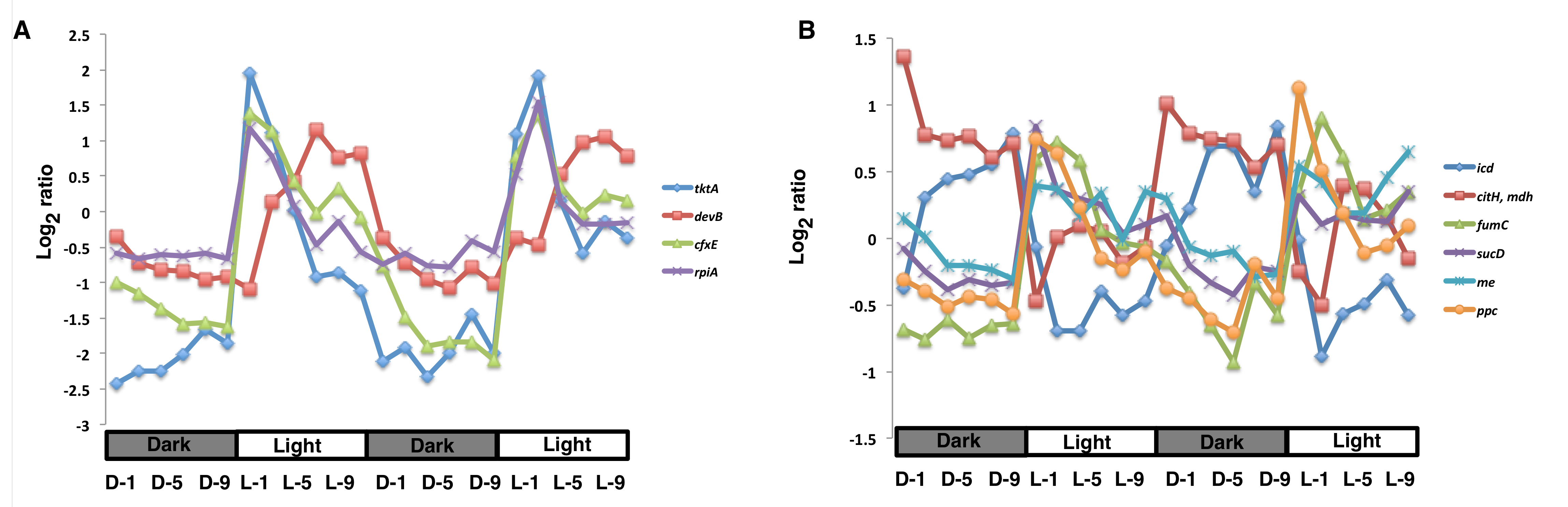

Supplement: Figure S4 — Expression profiles of genes with cyclic patterns that are involved in the OPPP (A) and the TCA cycle (B). L/D cycles are indicated as gray and white bars below the x axis, respectively. The log2 ratios of transcript abundance to the pooled sample control are plotted on the y axis. Download [file mbo002162797sf4.tif]

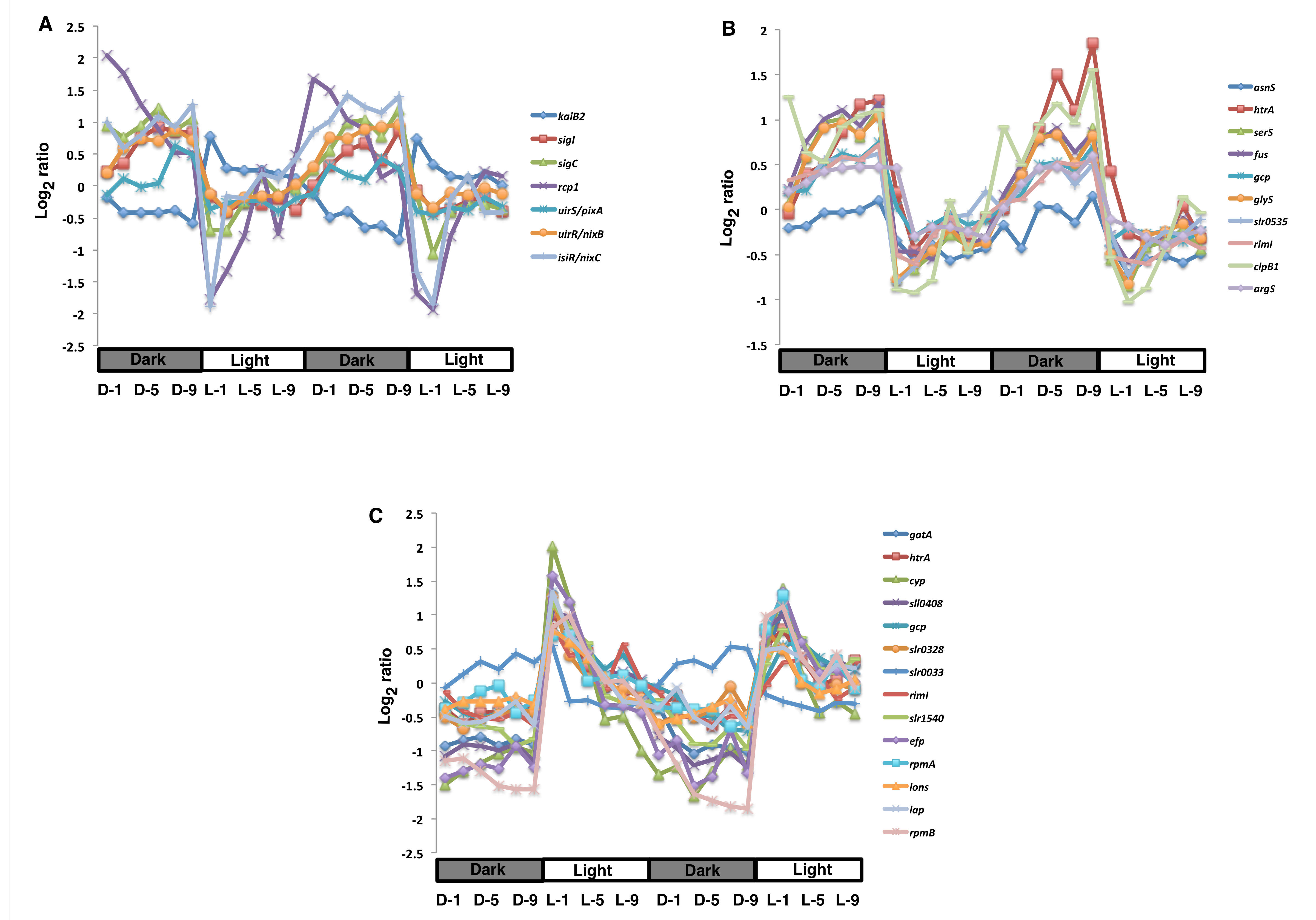

Supplement: Figure S5 — Expression profiles of genes with cyclic patterns that are involved in regulation (A), genes with maximal expression in the dark that are involved in translation (B), and genes with maximal expression in the light that are involved in translation (C). L/D cycles are indicated as gray and white bars below the x axis, respectively. The log2 ratios of transcript abundance to the pooled sample control are plotted on the y axis. Download [file mbo002162797sf5.tif]

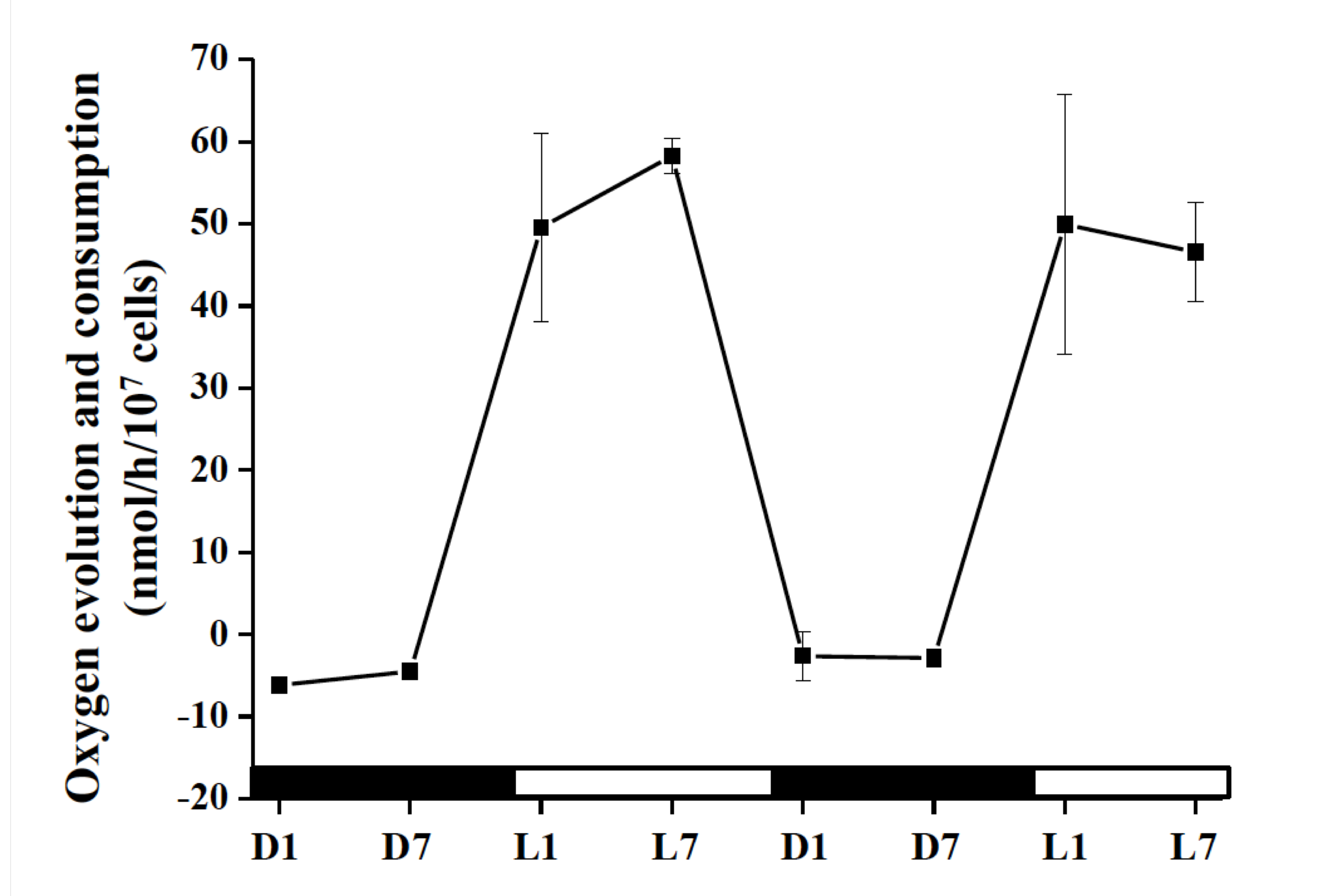

Supplement: Figure S6 — Oxygen production and consumption of Synechocystis. Samples collected at dark time points were always kept without any light to test respiration in the dark. Negative values represent oxygen consumption. Samples collected at light time points were tested with NaHCO3 as the electron acceptor to measure the oxygen production rate. Data are presented as the mean ± the standard deviation of three biological replicates. Download [file mbo002162797sf6.tif]
